# Predation Risk, and Not Shelter or Food Availability, as the Main Determinant of Reproduction Investment in Island Lizards

**DOI:** 10.3390/ani13233689

**Published:** 2023-11-28

**Authors:** Johannes Foufopoulos, Yilun Zhao, Kinsey M. Brock, Panayiotis Pafilis, Efstratios D. Valakos

**Affiliations:** 1 School for Environment and Sustainability, University of Michigan, Dana Building, 440 Church St., Ann Arbor, MI 48109, USA; 2 Geography and GIScience, University of Illinois, 1042 Natural History Building, Urbana, IL 61801, USA; 3 College of Natural Resources, University of California-Berkeley, Hilgard Hall, Wickson Rd., Berkeley, CA 94709, USA; 4 Museum of Vertebrate Zoology, University of California-Berkeley, Valley Life Sciences Building, 3101 UC Berkeley Rd., Berkeley, CA 94709, USA; 5 Department of Biology, Section of Zoology and Marine Biology, National and Kapodistrian University of Athens, Panepistimioupolis, Ilissia, 157-84 Athens, Greece; 6 Zoological Museum, National and Kapodistrian University of Athens, Panepistimioupolis, Ilissia, 157-84 Athens, Greece; 7 Department of Biology, Section of Animal and Human Physiology, National and Kapodistrian University of Athens, Panepistimioupolis, Ilissia, 157-84 Athens, Greece

**Keywords:** clutch size, clutch volume, islands, reproductive output, reptile

## Abstract

**Simple Summary:**

Understanding individual variation in reproductive investment, such as the number and size of eggs a mother produces in a single clutch, is an important species characteristic with implications for resilience to environmental change and conservation. In this study, we aimed to identify the main determinants of female reproductive investment in an island lizard species that experiences different local selection pressures depending on island size and local conditions such as vegetation cover, food availability, and predator species. We found that the number of eggs produced by a female is not just a function of maternal size, but is also strongly shaped by the richness of the local predator community: lizard populations living on islands with the fewest predators showed a >50% reduction in clutch size, as well as corresponding reductions in clutch volume. Our results emphasize the importance of local ecological conditions on vertebrate reproductive investment.

**Abstract:**

Reproductive investment, including the number of offspring produced, is one of the fundamental characteristics of a species. It is particularly important for island vertebrates, which face a disproportionate number of threats to their survival, because it predicts, among other things, a species’ resilience to environmental disruption. Taxa producing more offspring recover more quickly from environmental perturbations and survive environmental change better. However, ecologists do not understand which primary drivers shape a species’ reproductive investment well. Here, we compare the reproductive efforts of 14 island populations of the Aegean Wall Lizard (*Podarcis erhardii*), which lives across widely diverging environmental conditions. We test three hypotheses, namely that reproductive investment (measured as clutch size, clutch volume) is (1) positively associated with predation risk [‘Predation Risk Hypothesis’]; (2) positively associated with the presence of reliable vegetation cover that provides shelter [‘Gravid Female Protection Hypothesis’]; and (3) limited by (and hence positively correlated with) food availability [‘Food Limitation Hypothesis’]. Although field data are somewhat consistent with all three hypotheses, statistical analyses provide strong support for the Predation Risk Hypothesis. The results not only shed light on which fundamental forces shape reproductive investment in island vertebrates, but can also help shape conservation priorities.

## 1. Introduction

Island endemics inhabit only a relatively small area of the planet, yet they represent a significant component of Earth’s biodiversity. These taxa also represent a disproportionate fraction of Earth’s endangered biodiversity, with fully 41% of the world’s endangered terrestrial species found predominately in island systems [1]. Island species face particularly high extinction rates [2], as they are impacted by numerous threats including invasive organisms [3,4] and global climate change [5]. The rapidly deteriorating conservation status of island vertebrates adds urgency to understanding the endogenous and exogenous factors that drive their decline.

Island vertebrates are often characterized by a distinct suite of life history changes referred to under the umbrella term ‘island syndrome’ [6]. These changes have been observed across a broad range of disparate island organisms including mammals [7], birds [8], and reptiles [9]. Among reptiles, typical changes associated with island syndrome include shifts in body size [10,11,12], modified limb length and head shapes [13,14], and longer life spans and lower growth rates [15]. Island lizards tend to achieve higher densities [16,17,18], and may also display altered levels of intraspecific aggressiveness [12,19,20] as well as attenuated anti-predator morphologies and behaviors [21,22,23]. Probably the most widely recognized aspect of island syndrome is the modified pattern of reproductive investment, typically in the form of smaller clutches of larger eggs [24,25,26]. However, this pattern is neither universal [27,28] nor consistent [9], and it is not clear which factors drive reproductive investment.

Reproductive investment is a central aspect of a species’ life history and can vary tremendously even across closely related taxa [29,30,31]. Studies over the last half century have revealed a multitude of broad factors that can shape reproduction, including climate [32,33], latitude [30,34,35], elevation [36,37], foraging mode [38], phylogenetic history (e.g., [39,40], and general body bauplan [33], but also proximate environmental conditions such as refugium shape [41,42], resource availability [43], predation [44], and infection with parasites [45].

Chief among others, reproductive effort is thought to be subject to the constraints imposed by resource availability [43]. Numerous studies have demonstrated the importance of both condition and seasonal nutrient intake in shaping clutch size and clutch volume [28,46,47,48]. In practice, comparisons made between high and lower quality habitats or between high and low rainfall years have revealed that food availability can, but does not have to be, a driver of reproductive investment [43,49]. Moreover, it is not clear to what extend food availability modulates individual clutch size across years, and between individuals of a population, or whether it can also act as a long-term driver of macroevolutionary differences in reproductive output across different populations of a species.

Beyond resource availability, perhaps the most attention has been paid to the role of predation on reproductive investment. Both theoretical and empirical studies have revealed that predator-caused mortality can be a particularly important driver of an organism’s patterns of reproduction [38,50,51,52,53]. Indeed, according to classic life history theory, in areas of high mortality, selection should favor an early onset of reproduction and high reproductive investment, even if that comes at the cost of future reproduction [54]. Nonetheless, whether predator-induced mortality affects reproductive investment in relatively isolated island populations has been only rarely tested (e.g., [55]).

Numerous studies have shown that increases in reproductive investment in females come at the cost of declining running ability [56], and that this decline can also undermine longer-term survival [57], revealing a trade-off between current and future reproduction [58,59]. Gravid females can partially compensate for some of this loss in escape ability by modifying their foraging behavior [60,61,62] and initiating escape activities earlier [53,63,64]. Furthermore, gravid females stay closer to refugia [61,62] and reduce their visibility through appropriate microhabitat selection [60]. Consequently, habitats that lack or have only insufficient cover and refugia may exert selective pressure on reduced reproductive investment [65,66]. For example, in *Platynotus semitaeniatus,* an iguanid lizard species that seeks refuge in narrow rock crevices, females have evolved smaller clutches and a reduced clutch volume which, in turn, allow them to squeeze into smaller crevices to avoid predation [41,42]. Nonetheless, beyond this special case, the hypothesis that the availability of cover can affect reproductive investment has, to our knowledge, never been explicitly tested.

While a steadily growing body of literature has described patterns of vertebrate reproduction on islands, very few studies have investigated which are the proximate drivers that have shaped the evolution of these patterns. Because so many co-varying factors have the potential to affect reproductive output in wildlife [67], an increasing number of studies have taken an intraspecific approach and compared populations across a single widespread species [33,68,69,70]. By comparing multiple locations within the circumscribed geographic range of a single species, investigators can control for many confounding factors such as climate, elevation, body architecture, and phylogenetic effects and identify which microevolutionary processes are ultimately responsible for the observed variation in reproductive output. Focusing on island populations has the added benefit that they are evolutionarily discrete and, especially if small enough, can be considered homogenous in terms of prevailing environmental conditions.

In this study, we describe and analyze the evolutionary patterns of reproductive investment in the Aegean Wall Lizard (*Podarcis erhardii*)—a lacertid species widely distributed across the Aegean archipelago. By taking advantage of an unusual ecological setting where pronounced life-history differences exist between numerous well-characterized island populations, both in reproductive investment and in the prevailing ecological conditions, we test three fundamental hypotheses regarding the forces driving the evolution of clutch size in island taxa. Specifically, we test whether clutch size and clutch volume are determined by:

1. The amount of food available to the lizards (Food Limitation Hypothesis);
2. The amount of shelter available to the lizards (Gravid Female Protection Hypothesis);
3. The species richness of the local predator community (Predation Risk Hypothesis).

By elucidating these relationships, we aim to shed light on the drivers of the island syndrome and to understand the fundamental causes of reproductive investment in ectothermic organisms.

## 2. Materials and Methods

### 2.1. Study System

**Figure 1:**
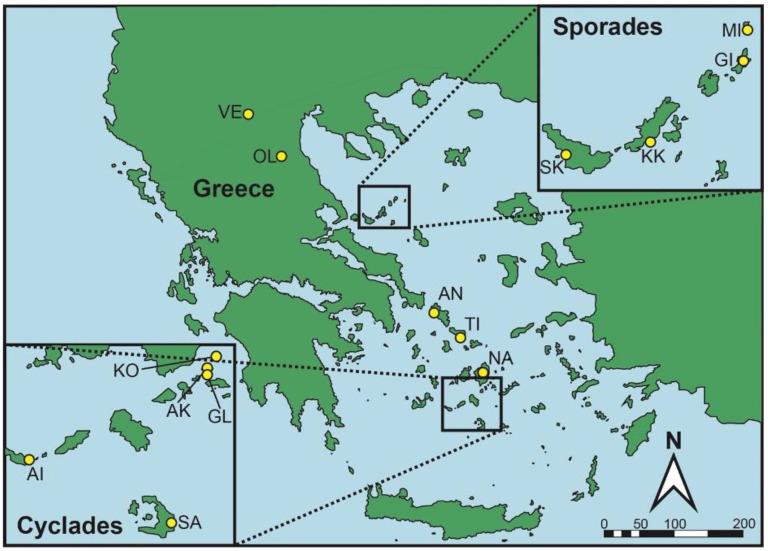
Map of the general area. Study sites are indicated by yellow circles and are identifiable by a two letter code: Agios Ioannis (AI), Andros (AN), Ano Koufonisi (AK), Gioura (GI), Glaronisi (GL), Kokkinonisi (KK), Kopria (KO), Mikropsathoura (Myga) (MI), Naxos (NA), Olympiada (OL), Santorini (SA), Skopelos (SK), Tinos (TI), Vevi (VE).

The study region lies predominantly in the Aegean Sea archipelago located between the southern Balkan peninsula in the west and the Anatolian mainland in the east. The study was conducted on 12 Aegean islands—8 islands in the Cyclades Cluster and 4 islands in the Sporades Cluster—as well as 2 sites located in nearby mainland Greece (Figure 1). Island sizes range from 0.005 km^2^ to 429 km^2^. The climate of the region is typically Mediterranean, with long, dry and warm summers and mild, rainy winters [71]. The vegetation cover on the study sites consists mostly of xerophytic, summer-deciduous, coastal heaths termed ‘phrygana’ (which are comprised of diverse, spinose plant communities rich in aromatic taxa), as well as of agricultural fields and sclerophyllous evergreen maquis [71]. The vegetation has been shaped by millennia of anthropogenic human activities, including terraced agriculture and small ruminant grazing [72].

### 2.2. Study Organism

The Aegean Wall Lizard (*Podarcis erhardii*) is a medium-sized lacertid lizard species with an adult Snout-Vent Length (SVL) of 49–78 mm [73]. This species is widely distributed across the Greek mainland and the Aegean Sea islands [74]. The species usually mates in spring and females lay their eggs in the period from April to July. Depending on the local conditions, eggs hatch in mid- to late summer [73]. Aegean wall lizards occur in a wide range of open habitats, with a preference for open, stony regions, and tend to be absent from areas with dense vegetation and close forest cover. They are particularly common in areas that provide refugia in the form of broken-up terrain and anthropogenic structures such as dry-stone walls and terraces [75]. *Podarcis erhardii* consume invertebreates opportunistically, eating a broad range of arthropods [76], with a particular emphasis on Coleoptera, Orthoptera, and soft-bodied larvae [77]. Additional secondary food items include snails [78] and even fruit. Observations suggest that the species displays at least occasionally cannibalistic tendencies [78,79].

### 2.3. Reproductive Traits

Morphological traits were obtained from museum specimens. Snout-vent length (SVL) and reproductive traits including clutch size, egg volume, and clutch volume were collected from specimens deposited at the Alexander König Zoological Research Museum in Bonn (Germany), as well as from the Zoologische Staatssammlung in Munich (Germany). While the species may occasionally lay a second clutch in mid- to late summer, museum collection dates indicate that all of the reproductive data reported here pertain to first clutches, and are therefore directly comparable across sites [77]. Following dissection, the number of oviductal eggs was recorded as clutch size (Table 1). We recorded the size of oviductal eggs, including the longest and shortest axes, using digital calipers. Egg volume was determined by using the equation for the volume (V) of an ellipsoid:

$$V=\frac{4}{3}\pi \alpha b^{2}$$

where α is half of the longest axis, and b is half of the shortest axis. The clutch volume for each female was calculated as the sum of the individual egg volumes [80].

### 2.4. Predation Pressure

Numerous types of predators feed on Aegean wall lizards, but their individual presence varies greatly across the range of *P. erhardii,* depending on ecological, vicariance, and biogeographic factors [21]. While mainland sites tend to harbor diverse communities of avian, mammalian, and reptile predators, some of the smallest islands fail to support even a single type of predator, hence creating a wide range of predation regimes. To obtain a more formal estimation of the predation environment that each population experiences, we followed the methodology of previous authors [21,23,55,81] and used predator species richness as a proxy of predation risk in each area. Predator presence data were obtained from the published literature and were then confirmed though our own field observations.

### 2.5. Measurement of Food Availability

We investigated the diet of *P. erhardii* across the study sites spanning a spectrum of ecological conditions by studying biomass and the abundance of arthropods in these sites during the key period during which females forage to form their clutches. Lizard food availability in the Aegean Sea can vary between years dependent on the extent of winter precipitation, and therefore can potentially obscure inter-island differences. However, empirical evidence suggests that this is not the case, as recent investigations have demonstrated that inter-island differences in food availability are sufficiently stable across different years to shape pronounced and consistent differences in *P. erhardii* body size.

From May to July 2017, five pitfall and sticky trap pairs were set on each island to collect crawling and flying arthropods. All pitfalls and sticky traps were set in randomly selected areas with natural vegetation in the immediate vicinity of the sites where the reproductive data specimens were collected. Specifically, crawling arthropod populations were analyzed by deploying five 400 mL pitfalls containing antifreeze. To determine flying insect populations, we set up five 15.24 cm × 30.48 cm sticky traps placed on 30 cm stakes, over or near the pitfalls on each island. Following collection, all arthropods from pitfalls were washed with ethanol to remove dirt and antifreeze and stored in 60 mL plastic wide-mouthed jars for subsequent identification and measurement. All arthropods were identified to order, and length was measured to the nearest mm using a ruler. The approximate biomass of each individual was then calculated using the standard length-to-biomass equation [82]:

$$W=0.0305\cdot L^{2.62}$$

The abundance and biomass of each sticky trap or pitfall trap was calculated and each island’s abundance and biomass were calculated by averaging each sticky strip or pitfall. Because the deployment time of the sticky strips and pitfalls from each island was different, the average abundance and biomass was standardized to a 48 h basis. 

### 2.6. Measurement of Vegetation

While Aegean Wall lizards will sometimes also use crevices in the rocky substrate as refugia, past and ongoing research has shown that the availability of brush cover is critically important for the survival of *P. erhardii* populations in most regions of the species’ range [77]. Hence, to assess the availability of such refugia, we quantified evergreen brush vegetation cover by utilizing a Normalized Difference Vegetation Index (NDVI) (“Measuring Vegetation”, NASA Earth Observatory). For each island, we downloaded Landsat 8 OLI/TIRS level 2 (surface reflectance) images from Earth Explorer, U.S. Geological Survey. To ensure the accuracy of NDVI, we used only images with less than 10% cloud cover. The NDVI of each surface reflectance image was calculated in ERDAS Imagine 2016. Each image was then input into ArcGIS 10 and clipped to the sample area with 62.5 m radius around each lizard collection site based on the size of the smallest of our field sites (Kokkinonisi). The NDVI of each pixel in the sample area was exported and for each island the NDVI of the sample area was calculated by averaging the values of the corresponding pixels.

### 2.7. Statistical Analyses

Former studies show that island size influences vegetation, arthropods, and predation risk [83,84,85]. To test this hypothesis, we used linear models to find the relationship between island size (island area) and the number of shelters (vegetation), food availability (biomass of arthropods), and predation risk (predator richness). Since island area has a highly skewed distribution, we used both island area and log-transformed island area as the independent variable when building linear regression models.

Because earlier research has shown that maternal SVL may affect reproductive traits, we included maternal SVL in some of the models as a covariate [28,86]. To avoid issues of collinearity, we tested the correlations between all explanatory variables considered in this paper (maternal SVL, NDVI, predator richness, and biomass of arthropods) and excluded any complex models that had variables that were highly correlated with each other (r > 0.5). Since multiple lizards from each island were measured and individuals from the same island are assumed to be more similar to each other, we built linear mixed effect models in addition to linear models to check the effect of shelter amount, predation risk, and food availability on reproductive traits.

Afterwards, Akaike information criterion (AIC) was used for the comparison of the models. For each reproductive trait, the same types of models (linear model or linear mixed effect model) were compared by AIC. The independent variable(s) in the model with the lowest AIC value were considered the most important factor(s) affecting reproductive traits. Data were analyzed in R and met all test assumptions.

## 3. Results

### 3.1. Linear Models of Island Size and Independent Variables

**Figure 2:**
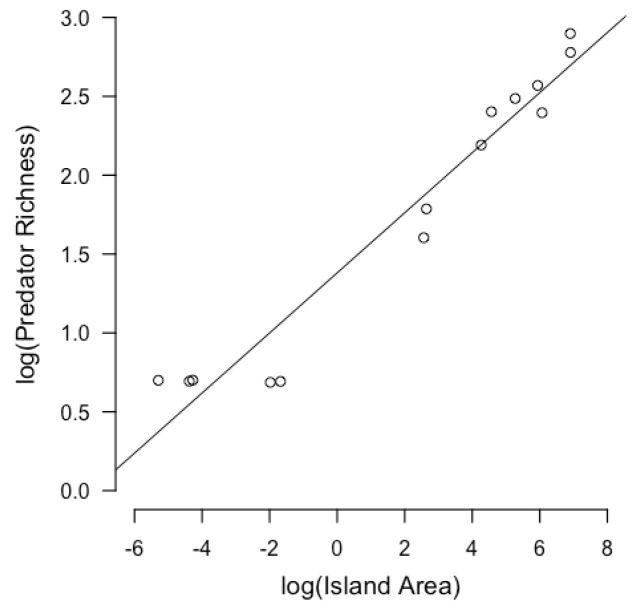
Linear regression between log-transformed island area and log-transformed predator richness. Each circle represents an island. Log-transformed predator richness has a positive relationship with log-transformed island area.

**Figure 3:**
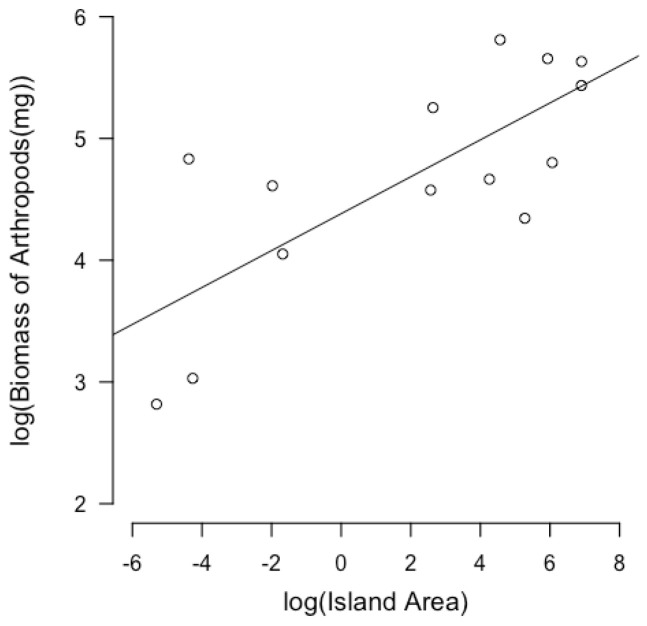
Linear regression between log-transformed island area and log-transformed biomass of arthropods. Each circle represents an island. Log-transformed biomass of arthropods has a positive relationship with log-transformed island area.

**Figure 4:**
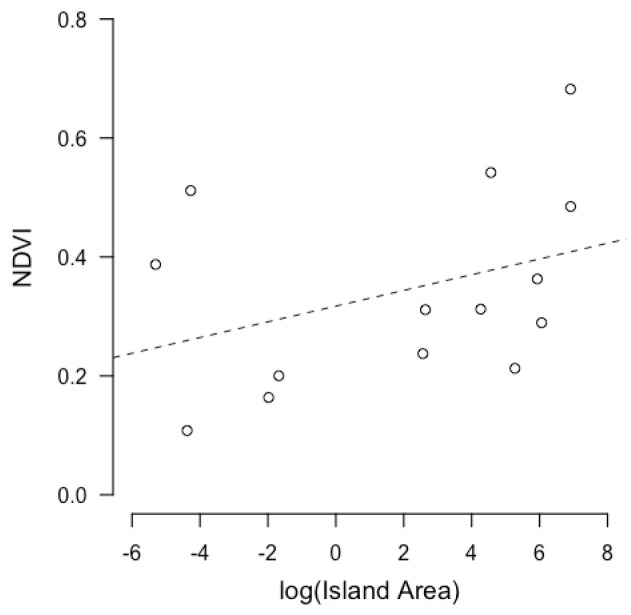
Linear regression between log-transformed island area and NDVI. Each circle represents an island.

The results of the linear models show that the log-transformed island area has a positive relationship with log-transformed predator richness (b = 0.190 ± 0.013, *p* < 0.001, R^2^ _adj_ = 0.942) (Figure 2). The log-transformed biomass of arthropods has a positive correlation with the log-transformed island area (b = 0.152 ± 0.038, *p* = 0.0018, R^2^ _adj_ = 0.531) (Figure 3). NDVI shows a positive relationship with island area (b = 0.0002752 ± 0.0001, *p* = 0.023, R^2^ _adj_ = 0.308) (Figure 4). Hence, larger islands tend to have higher predation risk, food availability, and extent of shelter-providing vegetation cover (Appendix A).

### 3.2. Correlations

The correlation between predator richness and NDVI is 0.650 (*p* < 0.001), the correlation between predator richness and biomass of arthropods is 0.701 (*p* < 0.001), and the correlation between arthropods biomass and NDVI is 0.600 (*p* < 0.001). Because the correlations among predator richness, biomass of arthropods, and NDVI are all higher than 0.5, resulting in potential collinearity issues, we did not include them as independent variables in the same models during the model building process. In contrast, the correlation between maternal snout-vent length and predator richness is 0.195 (*p* = 0.002); the correlation between maternal snout-vent length and NDVI is 0.102 (*p* = 0.111); and the correlation between maternal snout-vent length and the biomass of arthropods is −0.014 (*p* = 0.826), all precluding collinearity issues between these variables and maternal body size. Therefore, maternal snout-vent length and one of the other three independent variables were included in the same models.

### 3.3. Effect of Predator Richness, Biomass of Arthropods, and NDVI on Reproductive Traits

Among three reproductive traits, clutch size was found to be significantly related to predator richness (r = 0.087, *p* < 0.001, R^2^ _adj_ = 0.173), biomass of arthropods (r = 0.00341, *p* < 0.001, R^2^ _adj_ = 0.07464), and NDVI (r = 1.7898, *p* < 0.001, R^2^ _adj_ = 0.06778). Additionally, clutch volume had significant positive relationships with predator richness (r = 44.52, *p* < 0.001, R^2^ _adj_ = 0.0673) and biomass of arthropods (r = 1.659, *p* = 0.00828, R^2^ _adj_ = 0.02424). We also found significant positive effects of maternal snout-vent length on clutch size (r = 0.06146, *p* < 0.001, R^2^ _adj_ = 0.08326) and clutch volume (r = 30.07, *p* = 0.00505, R^2^ _adj_ = 0.0278) (Appendix B, Appendix C and Appendix D).

### 3.4. Hypothesis Testing

**Figure 5:**
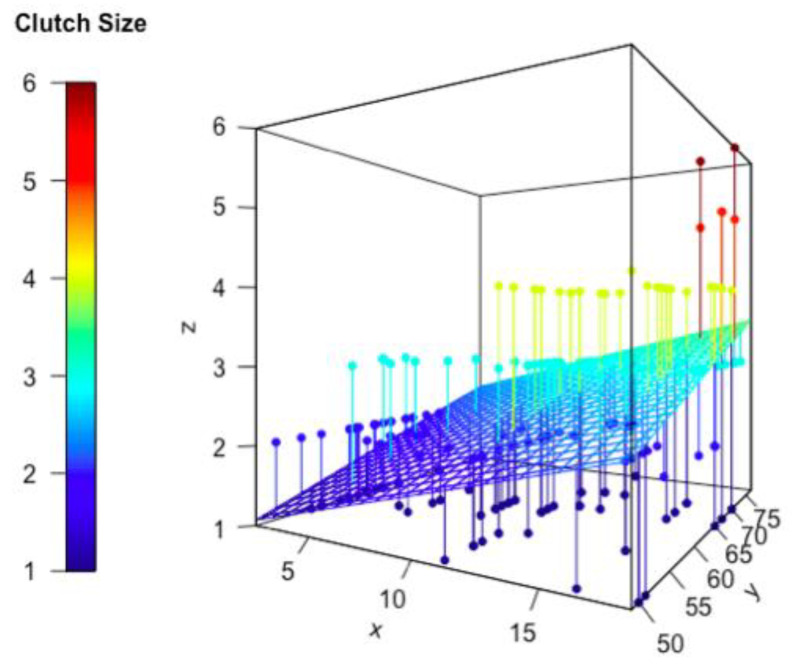
Lizard clutch size against predator species richness and maternal body size. Each dot represents one lizard clutch, and the wire mesh represents the predictions of the best model as provided from the AIC model comparison. x: Predator species richness, y: maternal snout-vent length (in mm), z: clutch size (number of eggs).

**Figure 6:**
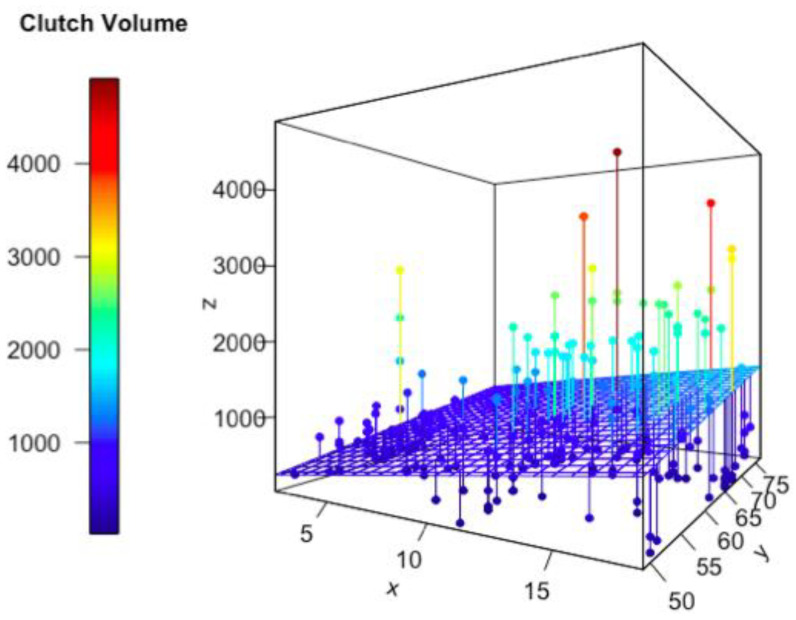
Lizard clutch volume against predator species richness and maternal body size (in mm). Each dot represents one lizard clutch, and the wire mesh represents the predictions of the linear model. x: Predator species richness, y: maternal snout-vent length (in mm), z: clutch volume (in mm^3^).

The results of AIC comparisons for the best mixed models are listed in Table 2 and Table 3 and Appendix E (runner-up linear models are in Table A1 and Table A2 in Appendix F). Among all linear mixed effect models for explaining clutch size, the model that contains predator richness and maternal SVL, and location as the random term, has the lowest AIC value (Table 2). For clutch volume, the linear mixed effect model including predator richness and maternal SVL as fixed terms, and location as the random term, has the lowest AIC value in terms of the highest explanation power (Table 3), although an alternative model that included NDVI, maternal SVL, and location was only marginally worse (Appendix E). Figure 5 and Figure 6 are the visualization of these two best models.

## 4. Discussion

Reproductive investment is a fundamental component of a species’ biology and has been the focus of many fruitful life-history studies [52,65,88,89]. It is also of practical importance; for example, a small clutch size has been shown to be a critical predictor of vulnerability to extinction in lizards [90]. Reptiles have emerged as particularly useful study systems to investigate the evolution of different reproductive investments because of the tremendous variety in reproductive modes that are made possible by ectothermy [30,91]. Because reproduction entails multiple conflicting demands and requires that organisms operate under limited resources (e.g., nutrients [43]; maternal body cavity volume [92]), it is impossible for an individual to optimize all aspects of its life history [30]. This, in turn, creates important trade-offs such as present-season versus future-season reproduction [50,54,57,93,94,95]. Other well-recognized trade-offs exist within a single clutch, e.g., the fundamental choice between the number of offspring and the size of individual offspring [34,96]. Ultimately, a lot of variation exists between as well as within species (e.g., [32,97]), and much of it remains unexplained.

In this study, we tested the effects of different factors on two different reproductive traits, clutch size and clutch volume. Our results provided support to the Predation Risk Hypothesis: we found that clutch size was significantly and positively correlated with predation risk; while food availability and vegetation cover also had a weaker relationship to clutch size, they had little explanatory power. Specifically, regarding clutch size, the model with the lowest AIC score included predator species richness and maternal snout-vent length as explanatory factors. Similarly, the marginally better model for clutch volume included predation risk and maternal snout-vent length as independent variables.

Our analyses indicate that the main factor associated with the evolution of different clutch sizes in island lizards is predator species richness (see Appendix G). As the species richness of syntopic predators decreases, so does clutch size, declining from an average of 2.9 eggs per clutch in Olympiada to 1.4 eggs per clutch on predator-poor Agios Ioannis. Similarly, predator species richness was the most important driver (albeit marginally) of clutch volume, with the smallest clutch volumes found on one of the most predator-poor islands (219 mm^3^, on Mikropsathoura). These results underscore the primary importance of predation pressure for Lacertid lizards and dovetail with recent investigations that highlight the importance of predation as a general driving force for the evolution of island lizards. For example, intraspecific comparisons across numerous island populations have demonstrated that wall lizards on reduced-predation islands have slower sprint speeds and tend to have relatively shorter legs [98,99]. Furthermore, lizards on low-predation islands have downregulated tail autotomy [100], stray further away from refugia [101,102], and are more apt to let potential predators approach before initiating escape behaviors [21,102] relative to lizards in predator-rich mainland regions.

Previous studies have argued that food availability is a critical driver of reproductive output in vertebrates [43,49,103,104]. While in temperate lizards and in species living in strongly seasonal environments, increased food availability generally translates into larger clutches [43], in more tropical or aseasonal environments it may instead result in more frequent clutches of the same size [49]. Although the Aegean Sea region is a strongly seasonal environment and *Podarcis* lizards tend to produce only one or at most two clutches annually [105], none of the models including food availability emerged as being the best at explaining the observed clutch size variation. While there were significant correlations between food availability and both clutch size and clutch volume, the explanatory power was very small (R^2^ _adj_ = 0.075 and 0.024, respectively). Part of this may be because the field methods did not allow us to adequately sample the preferred foods of the species. For example, *P. erhardii* appears to prefer to feed on larval Coleoptera and Orthoptera [106], two groups of arthropods that are hard to sample either in pitfall or in sticky traps. This species is also known to occasionally consume plant matter, but this does not make up a substantial part of its diet. Alternatively, it is known that clutch investment represents not just the nutrients available during the reproductive season that are then shunted towards reproduction, but also integrates stored lipids, and is a reflection of the general longer-term nutritional status of an animal rather than recent nutritional income. Lastly, it is possible that the average clutch size and volume are phylogenetically conservative traits that represent the long-term optimum for a population, rather than mapping tightly onto the local food availability in a given year. Either one of these may be the reason why we failed to detect a stronger relationship between food availability and clutch size or volume.

Numerous earlier studies have shown that increased reproductive output, whether in mammals [107], birds [108,109] or reptiles [110], incurs multiple costs that can affect future survival. Such costs stem from impaired thermoregulation [111], but especially from reduced locomotor performance, which in turn impairs the escaping ability from predators [53,63]. While the proximate drivers of reduced running speed in gravid females are complex [95], many of the costs center on the need to escape rapidly while carrying additional offspring mass along [110,111]. As a result, gravid females tend to stay close to hiding places, and the presence of sufficient cover and refugia can help females escape predation and perhaps thermoregulate more efficiently [99]. While *P. erhardii* generally prefers open areas, it requires the presence of hiding places such as sclerophyllous *phrygana* and maqui vegetation. Consequently, we predicted that the presence of cover in the form of evergreen shrubby vegetation, which can be measured as an island’s NDVI, would be positively related to a population’s reproductive investment. Instead, we found that there was only a weak relationship between clutch size and NDVI. We also found that there was some support for a model incorporating NDVI to explain clutch volume, although this model was not the best (Table 3).

## 5. Conclusions

In summary, when it comes to reproductive investment, wall lizard populations found in the Aegean Sea region constitute a textbook example of local adaptation. They showcase different degrees of expression of the island syndrome depending on the extend of insularity of a particular population. The number of eggs produced by a female is not just a function of maternal SVL, but is also strongly shaped by the richness of the resident predator community: lizard populations living on islands with the fewest predators showed a >50% reduction in clutch size, as well as corresponding reductions in clutch volume. At the same time, food availability appears to be a modest factor for both clutch size and clutch volume: lizards living on islands with higher food availability have more eggs and larger clutches. The presence of vegetation appears to be positively associated with clutch size (but not clutch volume), presumably because more plant cover allows for slower-moving gravid females to avoid being preyed on and perhaps because of thermoregulatory benefits. Our findings confirm and formalize a previous study highlighting the important effect of predation pressure on the reproductive output of other lizards in the Aegean Sea [55].

Traditional life history theory posits that the observed reductions in clutch size stem from a trade-off between clutch size and average egg (and therefore offspring) size. While in predator-rich environments selective pressures favor the production of a large number of small-bodied offspring, high intraspecific competition among young lizards in low-predation but high-lizard-density environments was expected to favor investment in large-bodied offspring [28]. Instead, we found that low-predation populations produce *both* small clutches and eggs with relatively unchanged size. There are at least two possible explanations for this pattern. First, it is possible that low-predation islands are also low-productivity environments that do not provide adequate resources for the production of large clutch numbers and volumes. Alternatively, it is possible that selection in low predation islands favors the evolution of long-lived life histories that produce small annual reproductive investment across many years. It is notable that these evolutionary patterns appear to have evolved several times, and relatively rapidly. Some of the strongest inter-population differences occur on neighboring islands (e.g., on Naxos and Glaronissi, which are <5 km away from each other and have been separated for <5000 years), indicating that such differences can evolve quite quickly.

## Figures and Tables

**Table 1:** Summary table including island name, coordinates, island size, predator richness, biomass of arthropods, NDVI, clutch size, and clutch volume (sample size).

Island Name	Coordinates	Island Size (km^2^)	Predator Richness	Biomass of Arthropods (mg)	NDVI	Clutch Size	Clutch Volume (mm^3^)
Kokkinonisi	39°9′38.757″ N, 23°54′7.129″ E	0.005	2	16.734	0.387	1.7 ± 0.3 (10)	598 ± 234 (10)
Mikropsathoura (Myga)	39°28′56.262″ N, 24°10′51.691″ E	0.014	2	20.724	0.511	1.6 ± 0.2 (11)	219 ± 60 (11)
Agios Ioannis	36°36′36.327″ N, 24°57′23.118″ E	0.033	2	125.727	0.108	1.4 ± 0.2 (10)	433 ± 143 (10)
Kopria	36°59′27.899″ N, 25°38′14.122″ E	0.138	2	100.870	0.164	1.6 ± 0.2 (10)	805 ± 254 (10)
Glaronisi	36°55′15.371″ N, 25°36′15.286″ E	0.188	2	57.548	0.200	1.9 ± 0.1 (8)	362 ± 100 (8)
Ano Koufonisi	36°56′49.45″ N, 25°36′20.237″ E	5.770	5	96.874	0.237	2.2 ± 0.2 (9)	386 ± 120 (9)
Gioura	39°23′46.899″ N, 24°10′20.407″ E	11.052	6	191.591	0.311	1.8 ± 0.3 (9)	549 ± 149 (9)
Santorini	36°22′59.326″ N, 25°28′29.843″ E	76.197	9	106.454	0.312	1.7 ± 0.1 (24)	612 ± 121 (24)
Skopelos	39°7′30.145″ N, 23°39′10.323″ E	96.229	11	333.154	0.542	2.5 ± 0.4 (12)	947 ± 235 (12)
Tinos	37°33′31.293″ N, 25°7′40.568″ E	194.500	12	77.232	0.213	2.7 ± 0.2 (23)	1268 ± 269 (23)
Andros	37°53′23.97″ N, 24°43′25.309″ E	380.000	13	286.327	0.363	2.2 ± 0.4 (9)	777 ± 289 (9)
Naxos	37°4′54.364″ N, 25°29′16.147″ E	429.785	11	121.837	0.289	2.4 ± 0.1 (42)	1008 ± 139 (42)
Olympiada	39°59′45.907″ N, 22°14′0.477″ E	1000.000	16	278.560	0.485	2.9 ± 0.2 (35)	1258 ± 175 (35)
Vevi	40°46′27.065″ N, 21°36′53.896″ E	1000.000	18	229.523	0.682	2.9 ± 0.2 (34)	951 ± 165 (34)

**Table 2:** AIC for seven linear mixed effect models constructed to explain clutch size.

Model	AICc	Δ AICc	Akaike Weight
CS ~ P + SVL + (1|Location)	714.703	0	0.687
CS ~ NDVI +SVL+ (1|Location)	716.997	2.293	0.218
CS ~ SVL + (1|Location)	719.623	4.920	5.871 × 10^−2^
CS ~ P + (1|Location)	720.750	6.047	3.342 × 10^−2^
CS ~ NDVI + (1|Location)	727.220	12.517	1.315 × 10^−3^
CS ~ B + SVL + (1|Location)	727.674	12.971	1.049 × 10^−3^
CS ~ B + (1|Location)	739.570	24.867	2.737 × 10^−6^

CS = clutch size, P = predator richness, B = biomass of arthropods, SVL = maternal snout-vent length. Models were ranked based on their AICc value differences. Associated Akaike weights are provided and were calculated based on the equation $W_{i}\left(AIC\right)=exp\left(-0.5\Delta_{i}\left(AIC\right)\right)/\overset{k}{\underset{k=1}{\sum }}exp(-0.5\Delta_{k}\left(AIC\right))$ [87].

**Table 3:** AIC for seven linear mixed effect models constructed to explain clutch volume.

Model	AIC	Δ AIC	Akaike Weight
CV ~ P + SVL + (1|Location)	4008.389	0	0.587
CV ~ NDVI + SVL + (1|Location)	4009.153	0.764	0.401
CV ~ P + (1|Location)	4017.407	9.018	6.461 × 10^−3^
CV ~ B + SVL + (1|Location)	4018.701	10.312	3.383 × 10^−3^
CV ~ NDVI + (1|Location)	4019.651	11.262	2.104 × 10^−3^
CV ~ SVL + (1|Location)	4022.031	13.642	6.401 × 10^−4^
CV ~ B + (1|Location)	4029.952	21.563	1.220 × 10^−5^

CV = clutch volume, P = predator richness, B = biomass of arthropods, SVL = maternal snout-vent length. Models were ranked based on their AICc value differences. Associated Akaike weights are provided and were calculated based on the equation $W_{i}\left(AIC\right)=exp\left(-0.5\Delta_{i}\left(AIC\right)\right)/\overset{k}{\underset{k=1}{\sum }}exp(-0.5\Delta_{k}\left(AIC\right))$ [87]. Random effects are indicated in parentheses.

## Data Availability

Data are available upon request from the authors.

## Appendix A

R outputs of linear regression between island area and independent variables. Significance notations: *—0.01 < *p* < 0.05; **—0.001 < *p* < 0.01; ***—*p* < 0.001. Same significance conventions apply to all subsequent Appendices.

## Appendix B

R outputs of linear regressions between clutch size and independent variables.

## Appendix C

R outputs of linear regressions between clutch volume and independent variables.

## Appendix D

R outputs of linear regressions between clutch size or clutch volume and maternal snout-vent length.

## Appendix E

R outputs of selected models to explain clutch size and clutch volume.

## Appendix F

AIC tables of linear mixed effect models of clutch size and linear models of clutch volume.

**Table A1:** AIC for seven linear models constructed to explain clutch size.

Model	AICc	∆ AICc	Akaike Weight
CS ~ P + SVL	695.691	0	0.998
CS ~ P	708.225	12.533	1.895 × 10^−3^
CS ~ B + SVL	712.715	17.024	2.007 × 10^−4^
CS ~ NDVI +SVL	719.651	23.959	6.257 × 10^−6^
CS ~ SVL	733.504	37.812	6.141 × 10^−9^
CS ~ B	735.805	40.114	1.943 × 10^−9^
CS ~ NDVI	737.624	41.933	7.826 × 10^−10^

CS = Clutch size, P = Predator richness, B = Biomass of arthropods, SVL = maternal snout-vent length. Models were ranked based on their AICc value differences. Associated Akaike weights are provided and were calculated based on the equation: $W_{i}\left(AIC\right)=exp\left(-0.5\Delta_{i}\left(AIC\right)\right)/\;\overset{k}{\underset{k=1}{\sum }}exp(-0.5\Delta_{k}\left(AIC\right))$ [87].

**Table A2:** AIC for seven linear models constructed to explain clutch volume.

Model	AIC	∆ AIC	Akaike Weight
CV ~ P + SVL	4030.073	0	0.753
CV ~ P	4032.505	2.432	0.223
CV ~ B + SVL	4037.205	7.132	2.128 × 10^−2^
CV ~ SVL	4042.707	12.634	1.359 × 10^−2^
CV ~ B	4043.606	13.533	8.668 × −10^−4^
CV ~ NDVI + SVL	4043.926	13.853	7.386 × 10^−4^
CV ~ NDVI	4049.325	19.252	4.967 × 10^−5^

CV = clutch volume, P = predator richness, B = biomass of arthropods, SVL = maternal snout-vent length. Models were compared with their AICc values and their associated Akaike weight, which was calculated using the following equation: $W_{i}\left(AIC\right)=exp\left(-0.5\Delta_{i}\left(AIC\right)\right)/\overset{k}{\underset{k=1}{\sum }}exp(-0.5\Delta_{k}\left(AIC\right))$ [87].

## Appendix G

**Table A3:** Predator and area information for each island. The numbers and names of predator species are given. Sources: (H&D) = G. Handrinos and A. Dimitropoulos. 1999. The Raptors of Greece (in Greek), Evstathiadis Group; (H&A) = G. Handrinos, Akriotis, F. 1997. The Birds of Greece. A&C Black (Publishers) Ltd., London, UK.; Cattaneo 1998. Gli Anfibi e i Rettili delle isole greche di Skyros, Skopelos e Alonissos (Sporadi settentrionali), Atti Soc. It. Sci. Nat. Museo Civ. Stor. Nat. Milano, 139/1998 (II): 127–149; (Chond) = Chondropoulos, B.P., 1989. A Checklist of Greek Reptiles. II. The Snakes. Herpetozoa, Wien, 2(1/2), pp. 3–36; JF—Johannes Foufopoulos, field obs.

Island Name	Area (km^2^)	Predators
Kokkinonisi	0.005	2 Snakes: — 0 Birds: *Corvus*, *F. eleon.* (H&D) Mammals: —
	
	
	
Mikropsathoura (Myiga)	0.014	2 Snakes: — 0 Birds: *Corvus* (Wt), *F. eleon.* (H&D) Mammals: —
	
	
	
Ag. Ioannis	0.033	2 Snakes: — 0 Birds: *Corvus*, *F. eleon.* (H&D) Mammals: — 0
	
	
	
Kopria	0.138	2 Snakes: — 0 Birds: *Corvus* Field observ. (JF) *F. eleon* (JF) Mammals:
	
	
	
Glaronisi	0.188	2 Rats: *F. eleon* (JF) Birds: *R. rattus* (JF)
	
	
Ano Koufonisi	5.770	5 Snakes: *Eryx* (JF), *Vipera* (JF). Birds: *F. tinnunculus* (JF), *Corvus* (JF) Mammals: *Rattus* sp.
	
	
Gioura	11.052	6 Snakes: *Colubridae* sp. (Legakis) Birds: *Corvus* (JF), *B. buteo*, *F. tinnunc* (H&D), *F. eleon* (H&D), Mammals: *Rattus* sp.
	
	
	
Santorini	76.197	9 Snakes: *E. sit* (Dimit), *E. quat* (Clarck 90), *T. fall* (Chon) Birds: *B. buteo* (H&D), *F. tinnunc* (H&D), *F. eleon* (H&D), *A. noct* (H&A), *L. sen* (H&A), *Corvus* (Wt), Mammals: *Rattus* sp.
	
	
	
Skopelos	96.229	11 Snakes: *E. sit* (Dimit), *M. monsp* (Dimit), *E. quat* (Cattan 98), *V. ammod* (Cattan) Birds: *Corvus* (Wt), *L. senat* (Wt), *B. buteo* (H&D), *F. tinnunc* (H&D) *F. eleon* (H&D), *A. noct* (H&A), *L. sen* (H&A) Mammals: *Rattus* sp., *M. foi* (JF)
	
	
	
Tinos	194.500	12 Snakes: *E. sit* (Dimit), *E. quat* (Dimit), *D. caspius* (Dimit), *N. natr* (Chon), *V. amm* (Chon), *T. fall* (Chon) Birds: *B. buteo* (H&D), *F. tinnunc* (H&D), *F. eleon* (H&D), *A. noct* (H&A), *L. sen* (H&A), *Corvus* (Wt), Mammals:
	
	
	
Andros	380.000	13 Snakes: *Z. sit*, *E. quat*, *D. casp*, *N. natr*, *T. fall*, *V. amm* Birds: *B. buteo*, *C. gall*, *L. sen*, *T. fall*
	
	
Naxos	429.785	Mammals: *R. rat*, *M. foi*, *V. vul* 11 Snakes: *E. quat* (JF), *N. natr* (Chon), *V. amm* (Chon) Birds: *Corvus* (Wt), *B. buteo* (H&D), *B. ruf* (H&A), *C. gallic* (H&A), *F. tinnunc* (H&D), *F. eleon* (H&D), *A. noct* (H&A), *L. sen* (H&A) Mammals: *R. rat*, *M. foi* (JF)
	
	
	
Olympiada	1000	16 Snakes: *Z. sit*, *E. quat*, *Z. long*, *D. casp*, *P. naj*, *N. natr*, *V. amm* Birds: *B. buteo*, *C. gall*, *C. aer*, *F. tinnunc*, *A. noct*, *L. coll*, *L. sen*, *Corvus* Mammals: *M. foi*
	
	
	
Vevi	1000	18 Snakes: *Z. sit*, *E. quat*, *D. casp*, *H. gem*, *N. natr*, *P. naj*, *M. monsp*, *V. ammo* Birds: *B. buteo*, *C. gall*, *C. aer*, *F. tinnunc*, *A. noct*, *L. coll*, *L. sen*, *Corvus* Mammals: *Rattus*, *M. foi* (JF)
	
	
	

Species abbreviations: Reptiles: *D. casp*—*Dolichophis caspius*; *E. quat*—*Elaphe quatuorlineata*; *N. natrix*—*Natrix natrix*; *T. fall*—*Telescopus fallax*; *V. amm*—*V. ammodytes*; *Z. sit*—*Zamenis situla*; *Z. long*—*Zamenis longissimus*. Birds: *A. noct*—*Athene noctua*; *B. buteo*—*Buteo buteo*; *B. ruf*—*Buteo rufinus*; *C. gall*—*Circaetus gallicus*; *C. aer*—*Circus aeroginosus*; *Corvus*—*Corvus corone* and/or *Corvus corax*; *F. eleon*—*Falco eleonorae*; *F. tinnunc*—*Falco tinnunculus*; *L. coll*—*Lanius collurio*; *L. sen*—*Lanius senator*. Mammals: *M. foi*—*Martes foina*; *R. rat*—*Rattus rattus*; *V. vulp*—*Vulpes vulpes*.
